# Variational Solutions and Random Dynamical Systems to SPDEs Perturbed by Fractional Gaussian Noise

**DOI:** 10.1155/2014/601327

**Published:** 2014-01-15

**Authors:** Caibin Zeng, Qigui Yang, Junfei Cao

**Affiliations:** ^1^School of Sciences, South China University of Technology, Guangzhou 510640, China; ^2^School of Automation Science and Engineering, South China University of Technology, Guangzhou 510640, China; ^3^Department of Mathematics, Guangdong University of Education, Guangzhou 510310, China

## Abstract

This paper deals with the following type of stochastic partial differential equations (SPDEs) perturbed by an infinite dimensional fractional Brownian motion with a suitable volatility coefficient Φ: *dX*(*t*) = *A*(*X*(*t*))*dt*+Φ(*t*)*dB* ^*H*^(*t*), where *A* is a nonlinear operator satisfying some monotonicity conditions. Using the variational approach, we prove the existence and uniqueness of variational solutions to such system. Moreover, we prove that this variational solution generates a random dynamical system. The main results are applied to a general type of nonlinear SPDEs and the stochastic generalized *p*-Laplacian equation.

## 1. Introduction

Recently, fractional Brownian motion (fBm) has been used successfully to model a variety of physical phenomena such as hydrology, turbulence, economic data, telecommunications, biology, and medicine. As a centered Gaussian process, it is characterized by the stationarity of its increments and the long-memory property. In general, the fBm represents a natural one-parameter extension (represented by the Hurst parameter *H*) of classical Brownian motion. It becomes the standard Brownian motion when *H* equals to 1/2. However, it was proved in [1] that the fBm is neither Markovian nor a semimartingale when *H* ≠ 1/2, which differs significantly from the standard Brownian motion. Thus, the well-established classical theory of stochastic analysis is not applicable to stochastic differential equations (SDEs) driven by fBm with *H* ≠ 1/2. This situation motivates a main mathematical challenge: how to extend the results in the classical stochastic analysis to fBm? Over the last years, some new techniques have been developed in order to define stochastic integrals with respect to fBm [2–10]. These techniques have the following common points: (1) they get harder as *H* gets smaller; (2) the more the paths of the stochastic process are irregular, the harder it is to integrate against them. Therefore, path regularity is a key benchmark to evaluate the mathematical tractability of any model with dependent noise.

On the other hand, the generation of a random dynamical system (or stochastic flow) from a stochastic partial differential equation (SPDE) is a fundamental problem in the study of its dynamics. It is well-known that a large class of partial differential equations (PDEs) with stationary random coefficients and Itô stochastic ordinary differential equations (ODEs) generate random dynamical systems (cf. the monograph [11]). However, stochastic equations driven by fBm do not generate a Markov process, which precludes the study of invariant measures for fBm-driven systems using classical tools. This motivates the study that fBm-driven SPDEs generate random dynamical systems. To the best of our knowledge, the generation of a random dynamical system (RDS) from a SPDE perturbed by general noise (in particular fBm) is far from fully solved. Nevertheless, some initial work has been done previously in this issue [12–14]. Under some regularity conditions, the asymptotic behavior of SPDEs driven by fBm was studied in [15, 16]. It should be pointed out that these papers were studied by using the semigroup (or mild solution) approach, for which it is necessary to have a linear operator in the drift part that has to generate a semigroup. There is also enormous research activity on nonlinear SPDEs since all kinds of dynamics with stochastic influence in nature or man-made complex systems can be modeled by such systems. In this case, variational approach has been used to investigate nonlinear SPDEs which are not necessarily of semilinear type. For more detailed examples, we refer the readers to [17, 18] and references therein. Within this framework, there seems to be only the work [19] analyzing the RDS from nonlinear SPDEs driven by infinite dimensional fBm. In [19], the existence of random attractors for a large class of SPDEs driven by general additive noise (including fBm) was established.

What is new in this paper is that we will add a suitable volatility coefficient for the infinite dimensional fBm. Based on the variational approach to SPDEs, we prove the existence and uniqueness of a variational solution to this general type of SPDEs perturbed by an infinite dimensional fBm with a suitable volatility coefficient, where the drift part is a nonlinear operator satisfying the standard monotonicity and coercivity conditions. Moreover, we show that this variational solution generates a continuous RDS.

This paper is organized as follows. In Section 2, we introduce some basic notations on RDS and stochastic integral with respect to fBm.  Section 3 contains the main results and the detailed proof. In Section 4, we apply the main results to two examples of SPDE including the stochastic generalized *p*-Laplacian equation with a suitable volatility coefficient for fBm.

## 2. Preliminaries

Throughout the paper, we assume that (*Ω*, *ℱ*, *ℙ*) is a probability space, *U* = (*U*, 〈·, ·〉_*U*_, ||·||_*U*_) is a separable Hilbert space, and *L* ^2^(*Ω*; *U*) stands for a space of all *U*-valued random variables *u* such that *𝔼*||*u*||^2^ = ∫_*Ω*_||*u*||_*U*_^2^ *dℙ* < *∞*.

### 2.1. Stochastic Integral for fBm

**Definition 1**

Let *H* ∈ (0,1). A Gaussian stochastic process *β* ^*H*^(*t*) is said to be a real-valued standard fractional Brownian motion (fBm) with Hurst parameter *H* if it satisfies that *𝔼β* ^*H*^(*t*) = 0 and

(1) $$\begin{array}{c} 𝔼\beta^{H}\left(t\right)\beta^{H}\left(s\right)=\frac{1}{2}\left({\left|t\right|}^{2H}+{\left|s\right|}^{2H}-{\left|t-s\right|}^{2H}\right),\;t,s\in \mathbb{R}. \end{array}$$

Assume that *Q* is a bounded linear and nonnegative symmetric operator on *U* and has finite trace; that is, there exists a complete orthogonal {*e*_*k*_}_*k*∈*ℕ*_ of *U* such that

(2) $$\begin{array}{c} Qe_{k}=\lambda_{k}e_{k},\;\mathrm{tr}Q=\overset{\infty }{\underset{k=1}{\sum }}\lambda_{k}<\infty ,\;\lambda_{k}\geq 0,\;\;k\in \mathbb{N}. \end{array}$$

Then, an infinite dimensional fBm *B* ^*H*^(*t*) with the incremental covariance operator *Q* is defined by

(3) $$\begin{array}{c} B^{H}\left(t\right):=\overset{\infty }{\underset{k=1}{\sum }}\sqrt{\lambda_{k}}\beta_{k}^{H}\left(t\right)e_{k}, \end{array}$$

where *β*_*k*_^*H*^(*t*) are independent real-valued fBms and the convergence in (3) holds *ℙ*-a.s. as well as in *U*. Throughout this paper, it is usually assumed that *H* ∈ (1/2,1). By the Kolmogorov continuity criterion (cf. [20, Theorem 1.4.1]), we have that *B* ^*H*^(*t*) has a continuous version since

(4) $$\begin{array}{c} 𝔼{||B^{H}\left(t\right)-B^{H}\left(s\right)||}_{U}^{2}=\mathrm{tr}Q{\left|t-s\right|}^{2H},\;t,s\in \mathbb{R}. \end{array}$$

Now, we recall stochastic integral [21] with respect to the fBms *β* ^*H*^(*t*) and *B* ^*H*^(*t*). For some fixed *T* > 0, let *ℰ* be the space of elementary functions:

(5) ℰ={f:f=∑i=1n−1fi1[ti,ti+1),  0=t1<t2<⋯<tn=T, fi∈ℋ},

where *ℋ* = (*ℋ*, 〈·, ·〉_*ℋ*_, ||·||_*ℋ*_) is another separable Hilbert space. For *f* ∈ *ℰ*, we define the stochastic integral

(6) $$\begin{array}{c} \overset{T}{\underset{0}{\int }}f\left(t\right)d\beta^{H}\left(t\right):=\overset{n-1}{\underset{i=1}{\sum }}f_{i}\left(t\right)\left(\beta^{H}\left(t_{i+1}\right)-\beta^{H}\left(t_{i}\right)\right). \end{array}$$

For *p* > 1/*H*, let *L* ^*p*^([0, *T*], *ℋ*) be the space of measurable functionas *f* : [0, *T*] → *ℋ* such that

(7) $$\begin{array}{c} {||f||}_{L^{p}([0,T],ℋ)}={\left(\iint_{0}^{T}{\langle f\left(t\right),f\left(s\right)\rangle }_{ℋ}\phi \left(t,s\right)dt\;ds\right)}^{1/2}<\infty , \end{array}$$

where *ϕ*(*t*, *s*) = *H*(2*H* − 1) | *t* − *s*|^2*H*−2^. From [22, Proposition 2.0.4], there exists some constant *C*_*p*,*T*_ only depending on *p* and *T* such that

(8) $$\begin{array}{c} 𝔼{||\overset{T}{\underset{0}{\int }}f\left(t\right)d\beta^{H}\left(t\right)||}_{ℋ}^{2}\leq C_{p,T}{||f||}_{L^{p}([0,T],ℋ)}^{2}. \end{array}$$

Then stochastic integral can be (almost surely) uniquely extended from *ℰ* to *L* ^*p*^([0, *T*], *ℋ*). Indeed it allows a continuous version due to the statement of [22, Proposition 2.0.8].

Now, we turn to the stochastic integral with respect to the infinite dimensional fBm. Denote by *L*_2_(*U*, *ℋ*) the family of Hilbert-Schmidt linear operators from *U* to *ℋ*. Let Φ : [0, *T*] → *L*_2_(*U*, *ℋ*) such that the function Φ(·)*u* is contained in *L* ^*p*^([0, *T*], *ℋ*) for each *u* ∈ *ℋ* and ||Φ||_*L*^*p*^([0,*T*],*L*_2_(*U*,*ℋ*))_ < *∞*. Then, we define the stochastic integral as

(9) $$\begin{array}{c} \overset{T}{\underset{0}{\int }}\Phi \left(s\right)dB^{H}\left(s\right):=\overset{\infty }{\underset{k=1}{\sum }}\sqrt{\lambda_{k}}\overset{T}{\underset{0}{\int }}\Phi \left(s\right)e_{k}d\beta_{k}^{H}\left(s\right), \end{array}$$

where the sum in right hand side of (9) converges absolutely in *L* ^2^(*Ω*; *ℋ*). Indeed, it, from [22, Lemma 2.0.10], follows that the stochastic integral (9) is an *ℋ*-valued Gaussian process.

### 2.2. Random Dynamical Systems

In this subsection, we recall some basic concepts and results on random dynamical systems that will be used to describe the dynamics of systems under the influence of a noise. For more details we refer the readers to the monograph [11].

**Definition 2**

A metric dynamical system (*Ω*, *ℱ*, *ℙ*, *θ*) with time ℝ consists of a measurable flow

(10) $$\begin{array}{c} \theta :\mathbb{R}\times \Omega ⟶\Omega \end{array}$$

which is (*ℬ*(ℝ) ⊗ *ℱ*; *ℱ*)-measurable and satisfies the flow property

(11) $$\begin{array}{c} \theta_{t_{1}}\circ \theta_{t_{2}}=\theta_{t_{1}}\theta_{t_{2}}=\theta_{t_{1}+t_{2}},\;\;\theta_{0}=id_{\Omega } \end{array}$$

for all *t*_1_, *t*_2_ ∈ ℝ. Additionally, we assume that the measure *ℙ* is invariant with respect to the flow *θ*.

Let *ℱ* be the associated Borel-*σ*-algebra. Then the operators *θ*_*t*_ forming the flow are given by the *Wiener shift*:

(12) $$\begin{array}{c} \theta_{t}\omega \left(\cdot \right)=\omega \left(\cdot +t\right)-\omega \left(t\right),\;t\in \mathbb{R}. \end{array}$$

According to [12, Theorem 2.3], we have the following conclusion.

**Remark 3**

Let *θ*_*t*_ be the flow of the Wiener shift and *ℙ* be the distribution of *B* ^*H*^(*t*), then the quadruple (*Ω*, *ℱ*, *ℙ*, *θ*) defines a metric dynamical system.

Furthermore, it holds that

(13) $$\begin{array}{c} B^{H}\left(\cdot ,\omega \right)=\omega \left(\cdot \right),\\ B^{H}\left(\cdot ,\theta_{r}\omega \right)=\omega \left(\cdot +r\right)-\omega \left(r\right)=B^{H}\left(\cdot +r,\omega \right)-B^{H}\left(r,\omega \right). \end{array}$$

We now recall the notion of random dynamical system.

**Definition 4**

A random dynamical system (RDS) with one-side time ℝ^+^ and phase space *ℋ* is a pair consisting of the metric dynamical system (*Ω*, *ℱ*, *ℙ*, *θ*) and a mapping

(14) $$\begin{array}{c} \varphi :{\mathbb{R}}^{+}\times \Omega \times ℋ⟶ℋ, \end{array}$$

which is (*ℬ*(ℝ^+^) ⊗ *ℱ* ⊗ *ℬ*(*ℋ*), *ℬ*(*ℋ*))-measurable and satisfies the cocycle property

(15) $$\begin{array}{c} \varphi \left(0,\omega ,\cdot \right)=id_{ℋ},\;\;\varphi \left(t+\tau ,\omega ,\cdot \right)=\varphi \left(t,\theta_{\tau }\omega ,\cdot \right)\circ \varphi \left(\tau ,\omega ,\cdot \right), \end{array}$$

for all *t*, *τ* ∈ ℝ^+^ and *ω* ∈ *Ω*. *φ* is said to be a continuous RDS if the mapping

(16) $$\begin{array}{c} x⟶\varphi \left(t,\omega ,x\right) \end{array}$$

is continuous for all *t* ∈ ℝ^+^ and *ω* ∈ *Ω*.

By (13) and [13, Lemma 5], we get the following property.

**Remark 5**

For *a*, *b*, *r* ∈ ℝ, it follows that

(17) $$\begin{array}{c} \overset{b}{\underset{a}{\int }}\Phi \left(s\right)d\omega \left(s\right)=\overset{b-r}{\underset{a-r}{\int }}\Phi \left(s+r\right)d\theta_{r}\omega \left(s\right). \end{array}$$

## 3. Main Results

Let *V*⊆*ℋ*⊆V* be a Gelfand triple; namely, *V* is a reflexive Banach space such that *V*⊆*ℋ* continuously and densely, *ℋ* is a separable Hilbert space defined in the previous section and *ℋ* ≡ ℋ* by the Riesz isomorphism. We consider the following stochastic partial differential equations (SPDEs):

(18) $$\begin{array}{c} dX\left(t\right)=A\left(X\left(t\right)\right)dt+\Phi \left(t\right)d\omega \left(t\right),\;X=x\in ℋ, \end{array}$$

where *ω* denotes a path of the infinite dimensional fBm *B* ^*H*^ with covariance function *Q* such that ∑_*k*=1_^*∞*^ *λ*_*k*_ < *∞*. Let *A* : *V* × *Ω* → V* be progressively measurable; that is, for *t* ∈ [0, *T*], *A* is (*ℬ*(*V*) ⊗ *ℱ*)-measurable, and Φ : [0, *T*] → *L*_2_(*U*, *ℋ*) such that the function Φ(·)*u* is contained in *L* ^*p*^([0, *T*], *ℋ*) for each *u* ∈ *ℋ* and ||Φ||_*L*^*p*^([0,*T*],*L*_2_(*U*,*ℋ*))_ < *∞*. Furthermore, we impose some conditions on *A* and Φ as follows.

 
*H*_1_ (*hemicontinuity*): for all *v*_1_, *v*_2_, *v* ∈ *V*, the map
  (19) $$\begin{array}{c} \lambda \;\;{⟼}_{V^{*}}{\langle A\left(v_{1}+\lambda v_{2}\right),v\rangle }_{V} \end{array}$$
 is continuous on ℝ.
 
*H*_2_ (*weak monotonicity*): there exists *c*_1_ ∈ ℝ such that for all *v*_1_, *v*_2_ ∈ *V*
  (20) $$\begin{array}{c} 2_{V^{*}}{\langle A\left(v_{1}\right)-A\left(v_{2}\right),v_{1}-v_{2}\rangle }_{V}\leq c_{1}{||v_{1}-v_{2}||}_{ℋ}^{2}. \end{array}$$
 
*H*_3_ (*coercivity*): there exist *c*_2_ > 0, *c*_3_ ∈ ℝ, *α* > 1, and an (*ℱ*_*t*_)-adapted process *f* ∈ *L* ^1^([0, *T*] × *Ω*, *dt* ⊗ *P*) such that for all *v* ∈ *V*,  *t* ∈ [0, *T*]
  (21) $$\begin{array}{c} 2_{V^{*}}{\langle A\left(v\right),v\rangle }_{V}+c_{2}{||v||}_{V}^{\alpha }\leq c_{3}{||v||}_{ℋ}^{2}+f\left(t\right). \end{array}$$
 
*H*_4_ (*boundedness*): there exist *c*_4_ ≥ 0 and an (*ℱ*_*t*_)-adapted process *g* ∈ *L* ^*α*/(*α*−1)^([0, *T*] × *Ω*, *dt* ⊗ *P*) such that for all *v* ∈ *V*,  *t* ∈ [0, *T*]
  (22) $$\begin{array}{c} {||A(v)||}_{V^{*}}^{}\leq g\left(t\right)+c_{4}{||v||}_{V}^{\alpha -1}. \end{array}$$
 
*H*_5_: there exists a finite dimensional subspace
  (23) $$\begin{array}{c} E=\mathrm{span}\left\{{\hat{e}}_{1},{\hat{e}}_{2},\ldots ,{\hat{e}}_{N}\right\}\subset V\left(\subset ℋ\right) \end{array}$$
 such that for the corresponding orthogonal projection *P*_*N*_ in *ℋ*, we have Φ(*t*) = *P*_*N*_Φ(*t*) for all *t* ∈ [0, *T*].

**Remark 6**

Noting that (*H*_1_)–(*H*_4_) are the standard monotonicity and coercivity conditions for SPDEs (cf. [18, Chapter 4]), on the other hand, assumption (*H*_5_) looks quite abstract at first glance. But it is very useful to deduce the following property. By *H*_5_ and [22, Corollary 2.0.16], we have that ||∫_0_^*t*^Φ(*s*)*dω*(*s*)||_*V*_^*α*^ and ||∫_0_^*t*^Φ(*s*)*dω*(*s*)||_*ℋ*_^*α*^ are all in *L* ^1^([0, *T*] × *Ω*, *dt* ⊗ *ℙ*), where *α* is as in *A*_3_. That is,

(24) $$\begin{array}{c} 𝔼\overset{T}{\underset{0}{\int }}{||\overset{t}{\underset{0}{\int }}\Phi (s)d\omega (s)||}_{V}^{\alpha }dt<\infty ,\\ 𝔼\overset{T}{\underset{0}{\int }}{||\overset{t}{\underset{0}{\int }}\Phi (s)d\omega (s)||}_{ℋ}^{\alpha }dt<\infty . \end{array}$$

**Definition 7**

A continuous *ℋ*-valued (*ℱ*_*t*_)-adapted process (*X*(*t*))_*t*∈[0,*T*]_ is called a variational solution to (18) if *X* ∈ *L* ^*α*^([0, *T*] × *Ω*, *dt* ⊗ *ℙ*; *V*)∩*L* ^2^([0, *T*] × *Ω*, *dt* ⊗ *ℙ*; *ℋ*) and

(25) $$\begin{array}{c} X\left(t\right)=x+\overset{t}{\underset{0}{\int }}A\left(X\left(s\right)\right)ds+\overset{t}{\underset{0}{\int }}\Phi \left(s\right)d\omega \left(s\right) \end{array}$$

holds for *t* ∈ [0, *T*], *ℙ*-a.s.

Now we claim and prove the main results.

**Theorem 8**

Suppose (*H*_1_)–(*H*_5_) hold. For any given *x* ∈ *L* ^2^(*Ω*; *ℋ*), there exists a unique variational solution to (18).

**Proof**

Let us define

(26) $$\begin{array}{c} Z_{t}\left(\omega \right)=\overset{t}{\underset{0}{\int }}\Phi \left(s\right)d\omega \left(s\right). \end{array}$$

Then for *t* ∈ [0, *T*] we get a new PDE

(27) $$\begin{array}{c} Y\left(t\right)=Y\left(0\right)+\overset{t}{\underset{0}{\int }}A\left(Y\left(s\right)+Z_{s}\left(\omega \right)\right)ds \end{array}$$

under the transformation *X*(*t*) = *Y*(*t*) + *Z*_*t*_(*ω*), where *Y*(0) = *x* ∈ *L* ^2^(*Ω*; *ℋ*). First, we show the existence and uniqueness of solutions to (27) by checking that ${\tilde{A}}_{\omega }(t,v)=A(v+Z_{t}(\omega ))$ satisfies conditions *H*_1_ to *H*_4_. Since *Z*_*t*_(*ω*) is an *ℋ*-valued Gaussian process, it is obvious to check the hemicontinuity and weak monotonicity for ${\tilde{A}}_{\omega }$ follow from that of *A*. For the coercivity, we also have that

(28) 2V∗〈A~ω(t,v),v〉V =2V∗〈A(v+Zt(ω)),v+Zt(ω)〉V  −2V∗〈A(v+Zt(ω)),Zt(ω)〉V ≤−c2||v+Zt(ω)||Vα+c3||v+Zt(ω)||ℋ2+f(t)  +2||A(v+Zt(ω))||V∗||Zt(ω)||V ≤−c221−α||v||Vα+c2||Zt(ω)||Vα+c3||v+Zt(ω)||ℋ2  +f(t)+2||A(v+Zt(ω))||V∗||Zt(ω)||V ≤−c221−α||v||Vα+c2||Zt(ω)||Vα+c3||v+Zt(ω)||ℋ2  +f(t)+2ε(α−1)α||A(v+Zt(ω))||V∗α/(α−1)  +2ε1−αα||Zt(ω)||Vα,

where the 1st inequality holds using *H*_3_, the 2nd inequality holds using that −||*v*+*Z*_*t*_(*ω*)||_*V*_^*α*^ ≤ −2^1−*α*^||*v*||_*V*_^*α*^ + ||*Z*_*t*_(*ω*)||_*V*_^*α*^, and the 3rd inequality holds using Young's inequality with a small value *ε* > 0. Next, by *H*_4_, we estimate

(29) ||A(v+Zt(ω))||V∗α/(α−1) ≤(g(t)+c4||v+Zt(ω)||Vα−1)α/(α−1) ≤21/(α−1)(g(t)α/(α−1)+c4||v+Zt(ω)||Vα) ≤21/(α−1)(g(t)α/(α−1)+c42α−1(||v||Vα+||Zt(ω)||Vα)).

Together (28) and (29), we get

(30) 2V∗〈A~ω(t,v),v〉V ≤−c221−α||v||Vα+c2||Zt(ω)||Vα+c3||v+Zt(ω)||ℋ2+f(t)  +2ε(α−1)α21/(α−1)  ×(g(t)α/(α−1)+c42α−1(||v||Vα+||Zt(ω)||Vα))  +2ε1−αα||Zt(ω)||Vα ≤−(c221−α−ε(α−1)α2(α2−α+1)/(α−1))||v||Vα+2c3||v||ℋ2  +(c2||Zt(ω)||Vα+2c3||Zt(ω)||ℋ2+f(t)    +2ε(α−1)α21/(α−1)    ×(g(t)α/(α−1)+c42α−1||Zt(ω)||Vα)     +2ε1−αα||Zt(ω)||Vα).

Let ${\tilde{c}}_{2}=c_{2}2^{1-\alpha }-(\varepsilon \left(\alpha -1\right)/\alpha )2^{\left(\alpha^{2}-\alpha +1\right)/\left(\alpha -1\right)}$, ${\tilde{c}}_{3}=2c_{3}$, and let $\tilde{f}(t)$ be the sum in the last parenthesis. Then we get

(31) $$\begin{array}{c} 2_{V^{*}}{\langle {\tilde{A}}_{\omega }\left(t,v\right),v\rangle }_{V}\leq -{\tilde{c}}_{2}{||v||}_{V}^{\alpha }+{\tilde{c}}_{3}{||v||}_{ℋ}^{2}+\tilde{f}\left(t\right). \end{array}$$

So we can pick *ε* to be sufficiently small such that ${\tilde{c}}_{2}>0$. Moreover, it follows from Remark 6 that ||*Z*_*t*_(*ω*)||_*V*_^*α*^ and ||*Z*_*t*_(*ω*)||_*ℋ*_^*α*^ are all in *L* ^1^([0, *T*] × *Ω*, *dt* ⊗ *ℙ*), and *g* ^*α*/(*α*−1)^ ∈ *L* ^1^([0, *T*] × *Ω*, *dt* ⊗ *ℙ*) holds due to *g* ∈ *L* ^*α*/(*α*−1)^([0, *T*] × *Ω*, *dt* ⊗ *ℙ*). Thus, $\tilde{f}\in L^{1}([0,T]\times \Omega ,dt⊗\mathbb{P})$. This completes the proof of the coercivity of ${\tilde{A}}_{\omega }$.

Indeed, by *H*_4_ we get

(32) ||A~ω(t,v)||V∗=||A(v+Zt(ω))||V∗≤g(t)+c4||v+Zt(ω)||Vα−1≤g(t)+c42α−1||Zt(ω)||Vα−1+c42α−1||v||Vα−1.

Let $\tilde{g}(t)=g(t)+c_{4}2^{\alpha -1}$ and ${\tilde{c}}_{4}=c_{4}2^{\alpha -1}$. Then we get

(33) $$\begin{array}{c} {||{\tilde{A}}_{\omega }(t,v)||}_{V^{*}}^{}\leq \tilde{g}\left(t\right)+{\tilde{c}}_{4}{||v||}_{V}^{\alpha -1}. \end{array}$$

It is easy to check that $\tilde{g}\in L^{\alpha /\left(\alpha -1\right)}([0,T]\times \Omega ,dt⊗\mathbb{P})$ since *g* ∈ *L* ^*α*/(*α*−1)^([0, *T*] × *Ω*, *dt* ⊗ *ℙ*) and ||*Z*_*t*_(*ω*)||_*V*_^*α*^ ∈ *L* ^1^([0, *T*] × *Ω*, *dt* ⊗ *ℙ*). Thus we prove the boundedness of ${\tilde{A}}_{\omega }$. Therefore, according to [18, Theorem 4.2.4], (27) has a unique solution

(34) $$\begin{array}{c} Y\in L^{\alpha }\left(\left[0,T\right]\times \Omega ,dt⊗\mathbb{P};V\right)\cap L^{2}\left(\left[0,T\right]\times \Omega ,dt⊗\mathbb{P};ℋ\right). \end{array}$$

Recall the transformation *X*(*t*) = *Y*(*t*) + *Z*_*t*_(*ω*), we have

(35) X(t)=x+∫0tA(Y(s)+Zs(ω))ds+Zt(ω)=x+∫0tA(X(s))ds+∫0tΦ(s)dω(s).

By Remark 6 we further have

(36) $$\begin{array}{c} X\in L^{\alpha }\left(\left[0,T\right]\times \Omega ,dt⊗\mathbb{P};V\right)\cap L^{2}\left(\left[0,T\right]\times \Omega ,dt⊗\mathbb{P};ℋ\right). \end{array}$$

Consequently, (18) has a unique variational solution in the sense of Definition 7.

The next theorem shows that the unique solution of (18) generates a random dynamical system *φ* in such a way that *φ*(*t*, *ω*, *x*) is defined by the solution of the SPDE at time *t*, for a noise path *ω*, with initial point *x*.

**Theorem 9**

The solution *X*(*t*, *ω*, *x*) of (18) defines a continuous random dynamical system *φ* : ℝ^+^ × *Ω* × *ℋ* → *ℋ* given by

(37) $$\begin{array}{c} \varphi \left(t,\omega ,x\right)=x+\overset{t}{\underset{0}{\int }}A\left(X\left(s\right)\right)ds+\overset{t}{\underset{0}{\int }}\Phi \left(s\right)d\omega \left(s\right). \end{array}$$

**Proof**

Trivially *φ*(0, *ω*, *x*) = *x*. Let us check then the cocycle property: for *t*, *τ* ∈ ℝ^+^ and *x* ∈ *ℋ*, we have

(38) φ(t+τ,ω,x)=x+∫0t+τA(X(s))ds+∫0t+τΦ(s)dω(s)=x+∫0τA(X(s))ds+∫0τΦ(s)dω(s)+∫τt+τA(X(s))ds+∫τt+τΦ(s)dω(s)=φ(τ,ω,x)+∫τt+τA(X(s))ds+∫τt+τΦ(s)dω(s).

Making the change of variable *r* = *s* − *τ* leads to

(39) $$\begin{array}{c} \overset{t+\tau }{\underset{\tau }{\int }}A\left(X\left(s\right)\right)ds=\overset{t}{\underset{0}{\int }}A\left(X\left(\tau +r\right)\right)dr. \end{array}$$

Applying Remark 5 we get

(40) $$\begin{array}{c} \overset{t+\tau }{\underset{\tau }{\int }}\Phi \left(s\right)d\omega \left(s\right)=\overset{t}{\underset{0}{\int }}\Phi \left(s+\tau \right)d\theta_{\tau }\omega \left(s\right). \end{array}$$

By the pathwise uniqueness of the solution to (27), we get

(41) $$\begin{array}{c} \varphi \left(t+\tau ,\omega ,x\right)=\varphi \left(t,\theta_{\tau }\omega ,\cdot \right)\circ \varphi \left(\tau ,\omega ,x\right). \end{array}$$

Now we turn to prove the measurability of *φ* : ℝ^+^ × *Ω* × *ℋ* → *ℋ*. Note that the maps *t* → *φ*(*t*, *ω*, *x*) and *x* → *φ*(*t*, *ω*, *x*) are continuous, thus we only need to prove the measurability of *ω* → *φ*(*t*, *ω*, *x*). By the proof of the existence and uniqueness of solutions to (18), we know that *φ*(*t*, *ω*, *x*) is the weak limit of a subsequence of the Galerkin approximations *φ* ^*n*^(*t*, *ω*, *x*) in *L* ^*α*^([0, *T*]; *V*). Let *ξ*_*k*_ ∈ *C*_0_^*∞*^(ℝ) be a Dirac sequence with supp(*ξ*_*k*_)⊆*B*_1/*k*_(0), where *B*_1/*k*_(0) is an open ball of radius 1/*k* centered at the point 0. Then (*ξ*_*k*_∗*φ* ^*n*^(·, *ω*, *x*))(*t*) is well defined for *k* large enough. For each such *k* ∈ *ℕ* and *h* ∈ *ℋ* we have

(42) $$\begin{array}{c} \left(\xi_{k}*{\langle \varphi^{n}\left(\cdot ,\omega ,x\right),h\rangle }_{ℋ}\right)\left(t\right)⟶\left(\xi_{k}*{\langle \varphi \left(\cdot ,\omega ,x\right),h\rangle }_{ℋ}\right)\left(t\right) \end{array}$$

when *n* → *∞*. Since *ω* → *φ* ^*n*^(·, *ω*, *x*) ∈ *L* ^*α*^([0, *T*]; *V*) is measurable, so is *ω* → (*ξ*_*k*_∗〈*φ* ^*n*^(·, *ω*, *x*), *h*〉_*ℋ*_)(*t*). Moreover, it follows from (42) that *ω* → (*ξ*_*k*_∗〈*φ*(·, *ω*, *x*), *h*〉_*ℋ*_)(*t*) is measurable. On the other hand, *t* → *φ*(*t*, *ω*, *x*) is continuous in *ℋ*. Therefore, (*ξ*_*k*_∗〈*φ*(·, *ω*, *x*), *h*〉_*ℋ*_)(*t*)→〈*φ*(·, *ω*, *x*), *h*〉_*ℋ*_(*t*) and the measurability of *ω* → (*ξ*_*k*_∗〈*φ*(·, *ω*, *x*), *h*〉_*ℋ*_)(*t*) imply the measurability of *ω* → 〈*φ*(·, *ω*, *x*), *h*〉_*ℋ*_(*t*). Since this is true for all *h* ∈ *ℋ*, we get the measurability of *ω* → *φ*(*t*, *ω*, *x*). Consequently, we complete the proof that *φ* defines a continuous RDS.

## 4. Application to Examples

In this section, we give two examples as application of the general results obtained in Theorems 8 and 9.

**Example 1**

Let 2 ≤ *p* < *∞*, Λ ⊂ ℝ^*d*^, Λ is open. Consider the following Gelfand triple:

(43) $$\begin{array}{c} V:=L^{p}\left(\Lambda \right)\subseteq ℋ:=L^{2}\left(\Lambda \right)\subseteq V^{*}:=L^{p/p-1}\left(\Lambda \right) \end{array}$$

and the SPDE

(44) $$\begin{array}{c} dX\left(t\right)=-X\left(t\right){\left|X\left(t\right)\right|}^{p-2}dt+\Phi \left(t\right)dB^{H}\left(t\right), \end{array}$$

where *B* ^*H*^ is an infinite dimensional fBm with Hurst parameter *H* > 1/2, and Φ is chosen as Section 3.

According to [18, Example 4.1.5], it is well known that (*H*_1_)–(*H*_4_) hold for *A*(*X*(*t*)) = −*X*(*t*) | *X*(*t*)|^*p*−2^ with *α* : = *p*. Thus, by the above results, for suitable Φ(*t*) the SPDE (44) has a unique variational solution taking values in *L* ^2^(Λ);, moreover, this solution defines a continuous RDS.

**Example 2 (stochastic generalized *p*-Laplacian equation)**

Again let 2 ≤ *p* < *∞*, Λ ⊂ ℝ^*d*^, Λ is open. Denote by *H*_0_^1,*p*^ the completion of *C*_0_^*∞*^(Λ) with respect to the norm defined as:

(45) $$\begin{array}{c} {||u||}_{1,p}:={\left(\int \left({\left|u\left(r\right)\right|}^{p}+{\left|\nabla u\left(r\right)\right|}^{p}\right)dr\right)}^{1/p}. \end{array}$$

Suppose Ψ : ℝ^*d*^ → ℝ^*d*^ is monotone, continuous and that for some strictly positive constants *a*_1_, *a*_2_

(46) $$\begin{array}{c} a_{1}{\left|u\right|}^{p-1}\leq \Psi \left(\left|u\right|\right)\leq a_{2}{\left|u\right|}^{p-1} \end{array}$$

for all *u* ∈ ℝ^*d*^. Then we consider the following Gelfand triple

(47) $$\begin{array}{c} V:=H_{0}^{1,p}\left(\Lambda \right)\subseteq ℋ:=L^{2}\left(\Lambda \right)\subseteq V^{*}:={\left(H_{0}^{1,p}\right)}^{*}\left(\Lambda \right) \end{array}$$

and the SPDE

(48) dX(t)=(div(Ψ(∇X(t)))−X(t)|X(t)|p−2)dt+Φ(t)dBH(t).

According to [22, subsection 5.4], it is well known that (*H*_1_)–(*H*_4_) hold for *A*(*X*(*t*)) = **d** **i** **v**(Ψ(∇*X*(*t*)) − *X*(*t*) | *X*(*t*)|^*p*−2^) with *α* : = *p*. Thus, by the above results, for suitable Φ(*t*), the SPDE (48) has a unique variational solution taking values in *L* ^2^(Λ); moreover, this solution defines a continuous RDS.

## 5. Conclusion

Using the variational approach we have studied a general type of fBm-driven nonlinear SPDEs with a suitable volatility coefficient in Hilbert space. We proved the existence and uniqueness of variational solutions to such system under some monotonicity and coercivity conditions. We further proved that this variational solution generates a random dynamical system. Finally, we applied the main results to two types of SPDEs including the stochastic generalized *p*-Laplacian equation. It is useful to note that our results for SPDEs in a Hilbert space can reduce to known results for a standard infinite dimensional Wiener process if the Hurst parameter *H* = 1/2 though the techniques of proof are different. Furthermore, the conditions (*H*_1_)–(*H*_4_) can be replaced by some much weaker assumptions (e.g., locally monotone) according to some recent results in [23]. When the nonlinear SPDEs are perturbed by a multiplicative fBm, it is interesting to study the random dynamical system from such more general nonlinear SPDEs by variational approach. This will be the subject for future investigation.
